# Evaluating the costs of developing a digital supervision tool for task-shared depression care: findings from a multi-site study in India

**DOI:** 10.1192/bjo.2026.12052

**Published:** 2026-08-10

**Authors:** Shreyas Kamat, Luanna Fernandes, Ankita Shah, Ravindra Agrawal, Anant Bhan, Chunling Lu, Abhijit Nadkarni, Carina Akemi Nakamura, Vikram Patel, Julia R. Pozuelo, Daisy Radha Singla, John A. Naslund

**Affiliations:** Addictions and Related Research Group, Sangath, Porvorim, India; Bhopal Hub, Sangath, Bhopal, India; Antarman Centre of Psychosocial Wellbeing, Panaji, India; Manipal Hospital Goa, Dona Paula, India; Division of Global Health Equity, Brigham and Women’s Hospital, Boston, Massachusetts, USA; Department of Global Health and Social Medicine, Harvard Medical School, Boston, Massachusetts, USA; Centre for Global Mental Health, Department of Population Health, London School of Hygiene & Tropical Medicine, London, UK; Department of Global Health and Population, Harvard TH Chan School of Public Health, Harvard Medical School, Boston, Massachusetts, USA; Department of Psychiatry, University of Oxford, Oxford, UK; Campbell Family Mental Health Research Institute, Centre for Addiction and Mental Health, Toronto, Canada; Department of Psychiatry, Temerty Faculty of Medicine, University of Toronto, Toronto, Canada; Department of Psychiatry, Lunenfeld-Tanenbaum Research Institute, Toronto, Canada

**Keywords:** Depression, task-sharing, economic evaluation, digital health, supervision

## Abstract

**Background:**

Task-sharing with non-specialist providers offers a promising solution to the mental health workforce shortage in low-resource settings. Supervision is essential to ensure treatment quality, but is often constrained by limited specialist availability.

**Aims:**

This study evaluated the costs of developing PEERS, a smartphone app that can facilitate registering and scheduling supervision sessions, collecting patient outcomes, rating therapy quality and assessing supervision quality among community-based non-specialist providers delivering behavioural activation for depression.

**Method:**

The PEERS digital application was developed between June 2021 and September 2022 for use in Madhya Pradesh and Goa, India. Development involved contributions from researchers, clinicians, technology experts and end users. Activity-based costing was used to systematically document the inputs and expenditures involved in developing the PEERS app and its accompanying training materials from a health systems perspective. Key cost components included human resources, leadership, IT support and infrastructure.

**Results:**

Total development costs were US＄130 027, with the costs of information technology accounting for the largest share (81%; US＄105 110). Human resource contributions accounted for 17% (US＄22 523), which included oversight and contributions from international research collaborators. Additional costs included overhead and infrastructure (US＄2393).

**Conclusions:**

Digital tools are increasingly being used to expand access to and support the delivery of mental health interventions in low-resource settings, yet few studies report on the development costs. By estimating the costs and resources required for developing a digital app for peer supervision, this study can inform efforts to facilitate broader implementation and adaptation of the app for use in other settings.

Given the global shortages of specialist mental health providers, task-sharing models have emerged as an evidence-based and effective strategy for delivering brief psychological interventions for a range of mental health conditions, including depression.^1,2^ Studies conducted in various countries, including India, have demonstrated the comparable effectiveness of leveraging non-specialist providers (NSPs) to deliver brief psychotherapy models.^1–4^ NSPs are individuals without specialised training in providing mental healthcare.^1,5^ To facilitate the successful delivery of high-quality psychotherapy for patients and to ensure continued support to this non-specialist workforce, ongoing supervision is a critical component.^6,7^ Best practices in task-sharing models involve coordinating regular supervision sessions, whereby an expert clinician observes or listens to audio-recorded treatment sessions and provides direct feedback on performance to NSPs.^5,8^ However, a central limitation with this model of supervision is the demands on the time of the few available expert clinicians, which is also expensive and limits scalability, especially in low-resource settings.^8^ Further, in-person supervision involves additional resources and logistics necessary to coordinate travel and scheduling.^9^ Thus, there is a clear need to explore alternative, scalable supervision models.

## Peer supervision

Peer supervision has emerged as an effective alternative to expert-led supervision, involving NSPs who have similar levels of training collaboratively supporting each other.^10^ Studies have shown that as the NSPs gain experience and competence, they achieve positive outcomes of the peer supervision process. Some of the outcomes measured include ability to provide reliable feedback, support self-care, support mutual learning from shared experiences and help with stress reduction.^11,12^ Building on this model, measurement-based peer supervision (MBPS) represents an innovative approach for objectively monitoring and evaluating the quality of delivery of psychological interventions, applied globally in low- and middle-income countries (LMICs)^1,13,14^ and high-income countries (HICs).^8,15,16^ MBPS utilises a psychometrically validated scoring framework to systematically assess quality of therapy delivery, drawing from audio-recorded treatment sessions, which are then shared with NSPs to obtain ratings from themselves, their peers and their supervisors or sometimes expert clinicians.^13^ Before supervision meetings, NSPs complete self-assessments, while peers (i.e. other NSPs in the group), and clinical experts also provide ratings. These ratings are then reviewed in an in-person or remote supervision session, during which narrative and qualitative feedback is also provided.^2,8,17^ In India, both lay and peer providers were able to rate their sessions as reliably as expert clinicians over time, and found this process to be highly acceptable and feasible.^13,14^ Thus, the MBPS model overcomes two significant limitations of conventional supervision methods: the long-term, overreliance on expert supervisors and logistical difficulties associated with conducting in-person meetings.^9^

## Use of digital technology

With the increased availability of smartphones and mobile internet, digital platforms offer a promising solution to overcome challenges of traditional in-person supervision.^18^ However, both digital mental health tools, as well as the interventionists’ skills and knowledge of using and integrating these tools in routine care, requires further development for successful adoption.^19^ A digital training programme for NSPs in India has already demonstrated considerable promise.^20–24^ Building on these successful efforts, the PEERS digital application was developed to facilitate remote peer supervision by enabling audio-recording of therapy sessions, followed by therapy quality rating, documentation and feedback.

## Relevance

There is a notable scarcity of research explicitly detailing the financial and resource-related requirements for creating digital tools aimed at facilitating the implementation of mental health interventions, particularly within LMICs.^25^ Detailed financial documentation is critical not only for transparency and accountability, but also for enabling informed decision-making and budgeting for adapting or modifying digital health initiatives for similar use in other resource-constrained settings.

The objective of this study was to comprehensively document the costs associated with developing the PEERS application. The PEERS mobile phone app (Version CommCare, 2.56.1 (471796)) is a proprietory application that was developed by Dimagi, India division, and is compatible with Android and iOS operating systems. Specifically, the study aimed to systematically record all resource expenditures, including human resources, leadership, infrastructure and information technology, involved in creating the PEERS digital application and any accompanying training materials and manuals (i.e. guides for training NSPs to use the PEERS app). By accurately capturing these costs, this study seeks to address existing gaps in knowledge regarding the financial implications of developing digital interventions aimed at supporting mental health service delivery involving task-sharing models within LMICs. Settings characterised by remote and underserved locations, with a scarcity of specialists and inequitable distribution of health services, stand to benefit from this study as they can effectively utilise this evidence to identify digital interventions that offer potential to transform the healthcare landscape.

The current study forms part of a larger project named PEERS (Promoting Effective Mental Healthcare through Peer Supervision) involving the systematic development, pilot testing and implementation of a digital application for peer supervision across multiple settings in India.^17^ This approach holds promise to streamline and enhance the supervision process, thereby supporting the implementation of task sharing and ensuring quality delivery of a brief psychological intervention for depression. To successfully replicate the use and adaptation of the PEERS app for diverse contexts, it is essential to document the resources and associated costs involved in its development.

## Method

### Design

This study involved a cost-analysis design, using a bottom-up approach to assess the economic costs and document the specific resources needed for developing a digital tool for facilitating peer supervision, a supervision manual and an e-course for the users of the digital application. All procedures performed in this study were approved by the relevant national and institutional committees, including ethical review boards at Sangath (EMPOWER: RA_2023_90; IMPRESS: AN_2017_033), Sinai Health (CTO 3614) and Harvard Medical School (IRB21-0992); adhere to Indian Council of Medical Research guidelines, 2017; and are in accordance with the Declaration of Helsinki of 1975 as revised in 2013. This study does not involve human participants, and informed consent was therefore not required.

### Setting

This costing study is part of the broader PEERS project,^17^ which is embedded within two larger implementation efforts: the EMPOWER (http://www.empowerindia.care/) initiative^16,26^ in Madhya Pradesh, India and the IMPRESS (Implementation of Evidence-Based Facility and Community Interventions to Reduce the Treatment Gap for Depression) trial in Goa, India (registered on ClinicalTrials.gov: NCT05890222).^27^

In EMPOWER Madhya Pradesh, the primary users of the PEERS supervision model and digital application are accredited social health activists (ASHAs), who serve as community health workers. ASHAs are female health workers deployed under India’s National Health Mission, operating at the community level in rural and underserved areas.^28^ As trusted members of the communities they serve, ASHAs play a vital role in delivering primary care services. Their work includes home visits, during which they typically provide preventive care, maternal and child health services, and support for chronic conditions. There has been growing interest in involving ASHAs in mental health services, as they are well-positioned to support community-level screening and intervention. Under the EMPOWER initiative,^16,26^ ASHAs in three rural districts of Madhya Pradesh, a central Indian state, were trained to identify symptoms of depression in their communities and deliver the Healthy Activity Programme (HAP), a brief psychological intervention based on behavioural activation for depression (described in detail below). They received supervision from clinical experts who were trained in HAP. Additionally, in one of these districts (Narmadapuram), they also received orientation to the PEERS supervision model and utilised the digital application for remote supervision.

The IMPRESS trial^27^ (registered on ClincalTrials.gov (NCT05890222.) on 12 May 2023) in Goa focuses on scaling up HAP delivery through the existing public healthcare infrastructure. As part of this initiative, a range of NSPs, including medical officers, staff nurses, multipurpose health workers and counsellors, were trained to deliver HAP and participate in supervision. These providers also received supervision from clinical experts and were oriented to PEERS as described above.

### HAP

HAP is a manualised, evidence-based behavioural activation intervention designed for delivery by NSPs to adults aged 18 years and over with moderately severe to severe depression, in primary care settings.^3,29,30^ HAP is typically offered to individuals with depressive symptoms as measured by a score of 10 or higher on the Patient Health Questionnaire-9, a brief tool used for screening and diagnosing severity of depression in adults.^31^ The core treatment components of HAP include psychoeducation, behavioural assessment, activity monitoring, activity scheduling and problem-solving. HAP is delivered individually over 6–8 weekly or fortnightly sessions, and sessions may take place at a primary care facility, the patient’s home or another preferred location.^3^ To ensure high-quality delivery of HAP, NSPs receive both individual and group supervision that provides essential clinical support and reinforces intervention fidelity. To strengthen this supervision process and enable remote supervision, the PEERS model and its accompanying digital application were developed.^17^ This system enables NSPs to effectively deliver HAP by ensuring structured support and allowing peer providers to evaluate therapy sessions using a psychometrically validated assessment tool.

### Development process: PEERS app

**Fig. 1:**
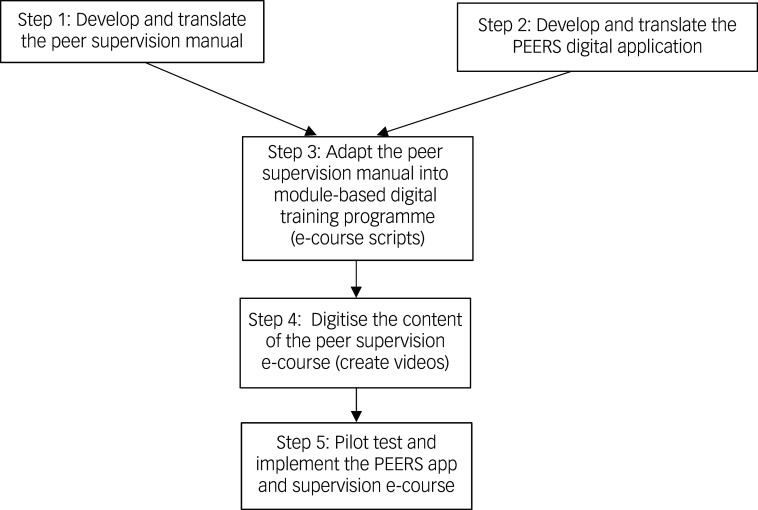
Steps for developing the PEERS app and accompanying training materials for non-specialists providers to deliver the Healthy Activity Programme for depression (from June 2021 to September 2022). PEERS, Promoting Effective Mental Healthcare through Peer Supervision.

The development of the PEERS app and accompanying supervision manual and training materials took place over a 16-month period, from June 2021 to September 2022. The key activities involved in the development process were divided into five sequential steps and detailed elsewhere.^17^ For the current study, we have briefly outlined these steps in Fig. 1 and summarised the development process in Supplementary File 1. In the sections that follow, we describe the steps involved in determining the costs and resources required for developing the PEERS app.

### Survey instrument development

We measured the costs of development from a health systems perspective. We applied the activity-based costing (ABC) approach to collect cost data in this study.^32^ ABC allocates costs based on the specific activities required to achieve the desired outcome. It operates on three core principles: outcomes are achieved through activities (Fig. 1), activities consume resources and resources incur costs.^32,33^ As this method records the activities and the resources required at each step of the development process, and then assigns the costs for each step, it is possible to extrapolate and estimate the costs of similar activities in another setting. For instance, this method prioritises the resources required for each activity rather than strictly the dollar amount, which can support replication of this work in other settings or at different time points where the dollar amounts could vary because of level of inflation.^33^ The ABC costing method has been extensively used in prior costing studies in the domain of public health in LMICs and other low-resource contexts.^33,34^ For this study, we used a bottom-up approach for cost data collection. We first mapped out the various development activities and then identified and assigned costs to each activity involved in the development of the PEERS app and supporting materials. This process is summarised in Table 1. The process of peer supervision does not involve patient participation. The PEERS app is a provider-facing app for the NSP, who uses the app to receive supervision. No costs were identified for patients or society at large, given that direct delivery of patient care was not an activity involved as part of developing the PEERS app.

**Table 1:** Cost-map of activities involved in development of the PEERS app Table 1 long description.

Mapping cost components for each activity				System-level costs				Patient-level costs	Community-level costs
Serial number	Activity	Time period (mmyy-mmyy)	Task	Human resources	Leadership	Infrastructure and logistical support	Technology		
1	Step 1: Develop the PEERS supervision manual	0621-0222	Draft manual	Yes	No	Yes	Yes	No	No
			Review manual	Yes	Yes	Yes	Yes	No	No
			Translate manual	Yes	No	Yes	Yes	No	No
2	Step 2: Develop the PEERS digital application	0621-0922	Meetings with Dimagi (IT vendor) team	Yes	No	Yes	Yes	No	No
			Internal user testing	Yes	No	Yes	Yes	No	No
			Identifying and resolving bugs	Yes	No	Yes	Yes	No	No
			Weekly meeting with collaborators	Yes	No	Yes	Yes	No	No
3	Step 3: Adapt the PEERS supervision manual into module based digital training programme (e-course scripts)	1221-0922	Draft e-course scripts	Yes	No	Yes	Yes	No	No
			Review e-course scripts	Yes	Yes	Yes	Yes	No	No
			Preparing slides	Yes	Yes	Yes	Yes	No	No
			Translation of slides and scripts	Yes	Yes	Yes	Yes	No	No
4	Step 4: Digitise the content of the PEERS supervision e-course (create videos)	0222-0922	Filming	Yes	No	Yes	Yes	No	No
			Editing	Yes	No	Yes	Yes	No	No
			Adding subtitles	Yes	No	Yes	Yes	No	No
			Animation	Yes	No	Yes	Yes	No	No
			Voiceovers	Yes	No	Yes	Yes	No	No
5	Step 5: Pilot test the PEERS app and supervision e-course in the field and get feedback	1021-0922	Field testing of the app	Yes	No	Yes	Yes	No	No
			Report issues and trouble-shooting technology difficulties	Yes	No	Yes	Yes	No	No
			Pilot e-course application	Yes	No	Yes	Yes	No	No
			Integrating feedback from health workers before implementation	Yes	No	Yes	Yes	No	No
			Preparing for focus group discussions/in-depth interviews	Yes	Yes	Yes	Yes	No	No
			Conducting focus group discussions/in-depth interviews	Yes	Yes	Yes	Yes	No	No
			Analysing data	Yes	Yes	Yes	Yes	No	No
			Revise app, manual, e-course post feedback	Yes	Yes	Yes	Yes	No	No
			Pilot testing, travel	Yes	Yes	Yes	Yes	No	No

PEERS, Promoting Effective Mental Healthcare through Peer Supervision; IT, information technology.

To structure the costing approach, we followed previous studies and adhered to the World Health Organization’s analytical framework for health systems,^35^ which comprises six building blocks: (a) health workforce, (b) leadership and governance, (c) financing, (d) health information systems, (e) service delivery and (f) access to essential medicines. We then collected costs of human resources (including leadership), infrastructure and information technology, which were the relevant domains for the context of this study. Costs related to each of these broad categories were mapped against the corresponding activities. A detailed mapping of these costs is outlined in Table 1. Furthermore, this study was guided by the activity map detailed in Fig. 1, and the alignment of costs with specific activities was developed in close consultation with key project stakeholders.

With this process, we developed survey instruments to capture project costs (see Supplementary File 2). The survey instruments recorded the resource name, its function, cost and duration of resource use. The survey templates, adapted from prior costing studies of a digital programme development in a similar setting in India and employed by our team, were tailored to the specific activities of this project.^25,36^ For human resources directly involved in and supervising activities, the survey captured the individual’s role, educational qualifications, average full-time equivalent, months worked, monthly salary, and travel and food reimbursements. For costs of the infrastructure, which consist of overhead costs, the instrument collected data on the total monthly cost of the utility, the total number of individuals using the utility and the number of individuals involved in the activity of interest (Fig. 1) to arrive at the proportionate costs. For costs of information technology, the survey instrument captured the total cost of the item, number of items, the useful life and the time duration for which the item was used for the activity of interest.

### Data collection

Time contributions were logged by individuals at the most granular level possible, ensuring the data closely reflected actual time spent on each activity. Infrastructure costs were sourced directly from project financial records, and overhead and device costs were calculated proportionately to their use for specific project activities (Fig. 1). Programme staff directly involved in activities completed monthly timesheets, documenting hours worked on each activity. They were trained to accurately identify and record the time contributed in the survey. All data were collected retrospectively, and site coordinators reviewed and verified their teams’ timesheets.^25^

Salary information for human resources directly involved in project activities was obtained from the official salary grades.^25^ For specialists (academicians and clinicians) who contributed expert review during the development process, time estimates were based on their participation in weekly meetings. Meeting minutes were reviewed to identify and quantify the time spent discussing the five steps of the development process (outlined in Fig. 1). Cost data related to information technology resources and infrastructural utilities used during development were extracted from financial records following the approach described in a previous study.^25^ The actual payments made to the external technology partner, Dimagi, for developing the digital application were recorded as outsourced development costs in the information technology cost category. These technology partner expenses reflect the total amount paid as part of a contract set up with the vendor. Infrastructure-related costs were also derived from financial records.

### Cost estimation and analysis

The cost of human resources was calculated by using the monthly number of hours spent on the activity of interest (Fig. 1), the number of months for which time was contributed and the monthly salary of the individual. Costs were calculated with time data collected through the survey instruments.^25^ To estimate the cost of expert input, information in the project budget or publicly available salary data for academics and clinicians from the respective countries were used to estimate costs.^37^

The annual value of equipment was calculated using the straight-line depreciation method, assuming zero salvage value.^25^ Proportional costs were then estimated based on the duration for which a device or service was used for specific development tasks. Infrastructure-related costs were allocated proportionally based on the number of human resources involved and the time spent on relevant activities. All cost data were initially collected in Indian Rupees (₹) and then converted to US Dollars by using an average 2021 exchange rate of US＄1 = ₹73.92. Additionally, values were converted to US＄ purchasing power parity (PPP) by using a rate of US＄1 PPP = ₹22.63. PPP is a conversion rate that shows the ratio of prices for a basket of goods in one currency compared with prices for the same basket of goods in another currency.^38^

## Results

The total cost of developing the PEERS app and the accompanying training materials was US＄130 027. A breakdown of these costs by major category is summarised in Table 2. The largest portion of this cost, approximately 81% (US＄105 110), was toward information technology. This included the payments made to the technology partner, Dimagi, for the design and development of the PEERS application. Given the intellectual and creative nature of the work, a share of 17% of the total cost (US＄22 523) was associated with human resources, including the clinical and academic experts involved in the intervention and app development process. These supervision-related expenses were primarily driven by contributions from researchers and experts from Canada, the UK, and the USA. In the sections that follow, we offer a breakdown of the various expenses within each cost category.

**Table 2:** Category-wise costs of developing the PEERS app and accompanying training materials Table 2 long description.

Cost category	IN₹	US＄^a^	US＄ PPP^b^	%
Human resources (including leadership)	1 664 937	22 523	73 572	17.32
Information technology	7 769 756	105 110	343 339	80.84
Infrastructure	176 871	2393	7816	1.84
Total	96 11 564	130 027	424 727	100

PEERS, Promoting Effective Mental Healthcare through Peer Supervision; PPP, Purchasing Power Parity.

a. US＄ = 73.918013 IN₹R in 2021.

b. US＄ PPP = 22.631556 IN₹ in 2021 (PPP is a conversion rate that shows the ratio of prices for a basket of goods in one currency compared with prices for the same basket of goods in another currency.^38^ IN₹ is the currency in the study settings.

### Information technology

Information technology costs accounted for the largest portion (approximately 81%) of the total development cost of the PEERS app, amounting to US＄105 110. These costs were driven by the payments made to the technology partner, Dimagi (US＄104 545), for the design and development of the digital application with custom features. We examined how the technology partner’s costs were distributed across different phases of app development between June 2021 and September 2022. The bulk of expenditures occurred between November 2021 and March 2022, coinciding with the release of version 1 of the PEERS app. The development process required use of devices such as laptops and mobile phones, as well as internet connectivity to support review meetings, digital content creation and pilot testing (Table 3).

**Table 3:** Itemised costs for information technology resources used in PEERS package development Table 3 long description.

Item	Cost IN₹	Cost US＄^a^	Cost US＄ PPP^b^
Technology partner (app developer costs)	7 727 966	104 545	341 492
Laptops	20 581	278	909
Mobile phones	1817	25	80
Internet	19 391	262	857
Total cost of information technology	77 69 756	1 05 110	3 43 339

PEERS, Promoting Effective Mental Healthcare through Peer Supervision; PPP, Purchasing Power Parity.

a. US＄ = 73.918013 IN₹ in 2021.

b. US＄ PPP = 22.631556 IN₹ in 2021 (PPP is a conversion rate that shows the ratio of prices for a basket of goods in one currency compared with prices for the same basket of goods in another currency.^38^ IN₹ is the currency in the study settings.

### Human resources

For the project/implementation side, human resources directly involved in the development activities (Fig. 1) and in leadership accounted for 17% (US＄22 523) of the total costs. Key contributors included costs for two intervention experts, one based at each site, who led the review of the peer supervision manual (step 1 in Fig. 1), and two project coordinators, who led the writing of the peer supervision manual and who were also actively involved in designing the digital application, developing the training materials, and conducting pilot testing and refinements. Additionally, two researchers handled the collection and synthesis of feedback following pilot testing of the PEERS app. Finally, seven peer supervisors participated in pilot testing and the development of the training materials.

This category also reflected the costs of study investigators and experts who supervised the process during the development of the PEERS app. Briefly, both local (*n* = 3) and international (*n* = 4) experts provided ongoing review and oversight throughout the development process. Costs related to leadership and oversight reflect the time contributions of these experts. The relatively high proportion of these costs in the overall budget is a result of the advanced skills, specialised knowledge and international experience of these individuals, four of whom are based in HICs. These experts played a key role by providing weekly supervisory input and feedback. Their time was mainly dedicated to developing the peer supervision manual and the digital application, which required extensive engagement. Developing the training materials and conducting pilot testing required comparatively less time from this group. The detailed responsibilities for each role, including both human resources directly involved in the activity and those involved in expert oversight, are described in Table 4.

**Table 4:** Itemised costs for human resources used in PEERS package development Table 4 long description.

Role/designation	Numbers	Responsibilities	Average FTE (%)	Total cost IN₹	Total cost US＄^a^	Total cost US＄ PPP^b^
Project coordinator(s)	2	Lead sites, draft peer supervision manual, internal user testing and field testing of app, identifying and resolving apps in the bug, drafting and reviewing e-course scripts, preparing slides for course modules, filming and editing module videos, translation of slides and scripts, voiceovers, make revisions to the PEERS app, manual and e-course post feedback	20.35	232 789	3149	139
Intervention coordinator(s)	2	Drafting and reviewing manual, internal and field testing of app, identifying and resolving bugs, drafting e-course scripts, review the translation of slides and scripts, Record voiceovers, filming, review the revision suggested for PEERS app and e-course post feedback	5.61	193 828	2622	116
Research assistant	2	Integrating feedback from focus group discussions, editing videos	8.04	6220	84	4
HAP supervisor	9	Field testing of PEERS app, draft and edit e-course scripts, preparing and translation of slides and scripts, voiceovers, subtitling, report issues in the PEERS app, revise manual, e-course post feedback	11.90	213 067	2882	127
Experts /collaborators (India and overseas)	6	Provide expert review on all aspects of the PEERS package, including the supervision manual, digital application and training e-course	0.45	306 819	4151	183
Principal investigator	1	Lead the project, provide overall guidance	5	712 213	9635	426
Total cost of human resources (including leadership)				1 664 937	22 523	995

PEERS, Promoting Effective Mental Healthcare through Peer Supervision; FTE, full-time equivalent; PPP, Purchasing Power Parity; HAP, Healthy Activity Programme.

a. US＄ = 73.918013 IN₹ in 2021.

b. US＄ PPP = 22.631556 IN₹ in 2021 (PPP is a conversion rate that shows the ratio of prices for a basket of goods in one currency compared with prices for the same basket of goods in another currency.^38^ IN₹ is the currency in the study settings.

### Infrastructure

This cost component represents expenses related to office rent and basic utilities used by the human resources involved in the project. Total infrastructure costs amounted to ＄2393, representing 2% of overall project costs (Table 5).

**Table 5:** Itemised costs for infrastructure resources used in PEERS package development Table 5 long description.

Item	Cost IN₹	Cost US＄^a^	Cost US＄ PPP^b^
Office space rental	1 45 814	1973	6443
Electricity	1369	419	1369
Total cost of infrastructure	1 76 801	2393	7813

PEERS, Promoting Effective Mental Healthcare through Peer Supervision; PPP, Purchasing Power Parity.

a. US＄ = 73.918013 IN₹ in 2021.

b. US＄ PPP = 22.631556 IN₹ in 2021 (PPP is a conversion rate that shows the ratio of prices for a basket of goods in one currency compared with prices for the same basket of goods in another currency.^38^ IN₹ is the currency in the study settings.

## Discussion

This study aimed to systematically record all resource expenditures, including human resources, leadership, infrastructure and information technology, involved in creating the PEERS digital application and any accompanying training materials and manuals (i.e. guides for training NSPs to use the PEERS app). This study offers a valuable contribution to the limited body of research on economics and costs related to digital health interventions, and specifically, development cost studies conducted in LMICs.^39^ Specifically, our study adds to the existing literature to extrapolate the costs of digital interventions in the context of mental healthcare delivery, where such studies are scarce and often dominated by data from HICs.^40^ The PEERS app is modifiable and adaptable to any other healthcare context, provider or intervention. The features of the app are generalisable to the delivery and supervision of other brief, manualised psychological interventions.^17^

Our study provides a detailed cost estimate for developing a comprehensive digital application, along with manual and training materials for enabling the peer supervision of NSPs delivering a brief psychological intervention for depression in community settings in India. The total development costs of the PEERS app were US＄130 027, with the majority (approximately 81% or US＄105 110) attributed to the information technology costs, reflected primarily as a contract paid to a vendor, toward designing and building the digital app. Additional costs included international expert leadership and contributions from the local field team, coordinators, researchers and clinical supervisors (17%, US＄22 523). These findings emphasise the crucial role of skilled human resources in developing digital health tools, another trend seen in related work, such as the 61% human resource share previously reported in creating a digital programme for training community health workers to deliver the same HAP intervention for depression care.^25^

The high proportion of costs dedicated to paying technology developers or vendors is consistent with other studies reporting the costs of digital health intervention development. For example, in a study that reported the costs of developing a web intervention, the Narrative Experiences Online Intervention (NEON) intervention, which provides recorded mental health recovery narratives to users, the costs of software development accounted for the largest component of overall costs.^41^ Another study evaluating the costs of developing and implementing an e-health intervention for children found that the cost of intervention material development (involving an animated show and a set of video games) was close to half of the total costs.^42^ Additionally, a study evaluating the costs of developing and implementing a digital intervention to prevent perinatal depression revealed that the percentage per patient costs of development with a tech company were the highest.^43^ We anticipate that the development of local capacities within LMICs, as well as with the recent advent and rapid growth of emerging technologies such as artificial intelligence (AI), there will be many opportunities to significantly reduce the costs of developing digital applications, and thereby reducing information technology costs in future efforts. For example, AI tools have already dramatically reduced the costs required for coding and software development, which yields new opportunities to adapt similar apps for a wide range of settings rapidly and with fewer resources required.

Notably, development costs for the PEERS app and its associated materials were also driven by the involvement of experts based overseas (i.e. outside of India). Future research could explore whether these roles might be fulfilled by local agencies or experts in LMICs and assess the potential effects of such local sourcing on both the cost and the quality of the final product. It would also be valuable to identify which specific skills, areas of expertise or types of experience are currently lacking in LMICs. These insights could assist stakeholders in LMICs in making strategic investments in local capacity building for future digital health innovations.

### Strengths

A strength with our study was drawing from use of an essential checklist previously employed in similar costing studies, which can ensure comparability across studies and promote transparency in reporting.^25^ We used a bottom-up costing approach that records the activities and the resources required at each step of the development process, and then assigns the costs for each step.^32^ This allows for extrapolation and estimation of the costs of similar activities in another setting. This approach recorded the resources required for each activity rather than only the costs in money terms, which can support replication of this work in other settings or at different time points where the dollar amounts could vary because of the level of inflation.^32,33^

This approach provides policy makers with more actionable insights by offering detailed information on the type, function and quantity of each resource, beyond simply reporting currency values. This is especially important given the wide variation in human resource costs between HICs and LMICs, and even within countries. Notably, the app was developed with NSPs with basic literacy and smartphone familiarity, suggesting strong potential for generalisability and scalability across diverse populations and settings. This study has relevance to a wider context in digital health initiatives. For example, there is ongoing work to adapt the PEERS app for use by community workers delivering brief psychosocial interventions in settings in Texas, USA.^15,44^

### Limitations

Several limitations of this study should be noted. First, the costs of expert leadership, which were estimated with information in the project budget, publicly available salary data and meeting minutes, may lead to over- or underestimation because of researcher bias. Additionally, the analysis only included costs incurred after the formal start of the grant, excluding pre-award efforts by the lead team, experts and technology partners, potentially resulting in underestimation. For overseas experts, overhead was estimated at 10% of salary costs, which aligns with the overheads permitted by the funder and may not reflect true overhead costs for each institution involved. Although most data were collected with a bottom-up approach, which is the ideal approach for costing digital intervention development, for the cost data gathered from our technology partner, we relied on periodic financial reports and contract details reflecting lumpsum payments for accomplishing project deliverables. Therefore, a more detailed analysis of the technology partner costs was not available in the current study, and such information would be warranted to ensure a more granular understanding of the project costs.

This study does not report the patient level costs of using the application. It focuses only on costs of development of the app, which did not involve patient participation. The PEERS app is a provider facing app for the NSP who uses the app to receive supervision. No costs were identified for patients or society at large, given that direct delivery of patient care was not an activity involved as part of developing the PEERS app. However, at the stage of implementation of this app, it would be relevant to measure the patient-level outcomes as well the costs to the patient. This is one avenue for exploration in future work.

Additional limitations include challenges in estimating costs when programme staff performed multiple roles, sometimes outside their formal training (e.g. voiceovers, video editing), potentially leading to overestimated time costs. Data collection for this study was done retrospectively introducing the possibility of recall bias. Asset costs were calculated with straight-line depreciation with zero salvage value; alternate methods could have yielded different results. Another limitation is the variation of prices used for various resources across different settings, which makes it challenging to generalise the cost estimates beyond the study sites. Information on resources used, recorded by this study, is therefore more important to the policy makers or other stakeholders in other countries and settings, as these details can directly guide what materials and personnel would be required to complete similar deliverables as this project. Although this study documents the costs of development of a digital app for peer supervision, the costs of scaling up, implementation and regular updates to such an app are important pieces of information for policy makers, and will require a separate analysis.

To summarise, digital technologies hold significant promise for training and supporting NSPs in delivering proven interventions for mental health challenges across diverse settings.^18^ Although this study documents the costs of development of a digital app for peer supervision, the costs of scaling up and implementation of such an app are an important component to provide a complete picture. For policy makers and funders, understanding the true costs of developing and implementing these solutions is essential for making informed decisions about resource allocation. Digital applications are inherently dynamic and often require iterative updates and refinements based on user feedback and technological advancements.^45^ Moreover, suboptimal adoption is frequently linked to incomplete cost planning and inadequate training for end users.^46^ Therefore, it is critical to assess separately the costs associated with training NSPs to employ the PEERS app in their work and the overall implementation and use of the PEERS app for facilitating supervision. In this context, a digital solution for peer supervision represents a meaningful step toward advancing task-sharing models and reducing the mental health treatment gap in LMICs.

This study offers a critical contribution to the limited evidence based on the costs of developing digital interventions for use in mental health care delivery in LMICs. As digital technologies increasingly play a central role in scaling evidence-based care,^18^ there is an urgent need for policy makers and funders to understand the true resource requirements for designing, deploying and sustaining these innovations. Our findings highlight that human expertise and technical development remain the most significant cost drivers. Our study also underscores the importance of systematically documenting the development costs and mapping the specific resources required to inform strategic investments and to support sustainable digital health systems. These insights are essential to guide future efforts to deliver scalable, equitable and high-quality mental healthcare across diverse low-resource settings in India and globally.

PEERS, Promoting Effective Mental Healthcare through Peer Supervision.

## Supporting information

10.1192/bjo.2026.12052.sm001 Kamat et al. supplementary material 1 Kamat et al. supplementary material

10.1192/bjo.2026.12052.sm002 Kamat et al. supplementary material 2 Kamat et al. supplementary material

## Data Availability

The data that support the findings of this study are available from the corresponding author, D.R.S., upon reasonable request.

## Supplementary material

The supplementary material is available online at https://doi.org/10.1192/bjo.2026.12052

## Table 1 Long description

A table mapping cost components for activities involved in developing the PEERS app. The table has 7 rows and 7 columns. The columns are labeled ‘Sr no’, ‘Activity’, ‘Time period (mmyy-mmyy)’, ‘Task’, ‘Human resources’, ‘Infrastructure and logistical support’, ‘Technology’, ‘Patient-level costs’, and ‘Community-level costs’. The rows detail various steps and tasks involved in the development process, including drafting, reviewing, translating manuals, developing digital applications, adapting manuals into digital training programs, digitizing content, and pilot testing the app. Each row specifies the time period, tasks, and the resources required for each activity, indicating whether human resources, infrastructure, technology, patient-level costs, and community-level costs are involved.

Navigate back to Table 1.

## Table 2 Long description

The table presents the category-wise costs of developing the PEERS app and accompanying training materials. It has four rows and four columns. The columns are labeled Cost category, IN, US＄, and % PPP. The row labels are Human resources (including leadership), Information technology, Infrastructure, and Total. The values in the table are as follows: Row 1: Human resources (including leadership), 1 664 937, 22 523, 73 572, 17.32%. Row 2: Information technology, 7 769 756, 105 110, 343 339, 80.84%. Row 3: Infrastructure, 176 871, 2393, 7816, 1.84%. Row 4: Total, 96 11 564, 130 027, 424 727, 100%. The table shows that the largest portion of the cost was towards information technology, followed by human resources, and a smaller portion towards infrastructure.

Navigate back to Table 2.

## Table 3 Long description

A table comparing costs of information technology resources for app development. The table has four rows and three columns. The columns are labeled ‘Item’, ‘Cost IN’, ‘Cost US＄’, and ‘Cost ＄ PPP’. The rows are labeled with different items and their corresponding costs. Row 1: Technology partner (app developer costs), 7 727 966, 104 545, 341 492. Row 2: Laptops, 20 581, 278, 909. Row 3: Mobile phones, 1817, 25, 80. Row 4: Internet, 19 391, 262, 857. Row 5: Total cost of information technology, 77 69 756, 1 05 110, 3 43 339.

Navigate back to Table 3.

## Table 4 Long description

A table detailing the costs for human resources used in PEERS package development. The table has 10 rows and 6 columns. The columns are labeled Role/designation, Numbers, Responsibilities, Average FTE, Total cost IN ＄, and Total cost PPP. The rows list different roles and their respective details. Row 1: Project coordinator(s), 2, Lead sites, draft peer supervision manual, internal user testing and field testing of app, identifying and resolving apps in the bug, drafting and reviewing e-course scripts, preparing slides for course modules, filming and editing module videos, translation of slides and scripts, voicers, make reviews to the PEERS app, manual and e-course post feedback, 20.35, 232 789, 3149. Row 2: Intervention coordinator(s), 2, Drafting and reviewing manual, internal and field testing of app, identifying and resolving bugs, drafting e-course scripts, review the translation of slides and scripts, Record voicers, filming, review the revision suggested for PEERS app and e-course post feedback, 5.61, 193 828, 2622. Row 3: Research assistant, 2, Integrating feedback from focus group discussions, editing videos, 8.04, 6220, 84. Row 4: HAP supervisor, 2, Field testing of PEERS app, draft and edit e-course scripts, preparing and translation of slides and scripts, voicers, subtitling, report issues in the PEERS app, revise manual, e-course post feedback, 11.90, 213 067, 2882. Row 5: Experts /collaborators (india and overseas), 6, Provide expert review on all aspects of the PEERS package, including the supervision manual, digital application and training e-course, 0.45, 306 819, 4151. Row 6: Principal investigator, 1, Lead the project, provide overall guidance, 5, 712 213, 9635. Row 7: Total cost of human resources (including leadership), 16, 1 664, 22 523, 995.

Navigate back to Table 4.

## Table 5 Long description

A table comparing the costs of office space rental and electricity for infrastructure resources used in PEERS package development. The table has three rows and four columns. The columns are labeled Item, Cost IN, Cost US＄, and Cost PPP. The rows are labeled Office space rental, Electricity, and Total cost of infrastructure. Row 1: Office space rental, Cost IN 145814, Cost US＄ 1973, Cost PPP 6443. Row 2: Electricity, Cost IN 1369, Cost US＄ 419, Cost PPP 1369. Row 3: Total cost of infrastructure, Cost IN 176801, Cost US＄ 2393, Cost PPP 7813.

Navigate back to Table 5.
