# Supplementary material for: Evaluating the costs of developing a digital supervision tool for task-shared depression care: findings from a multi-site study in India
Source: BJPsych Open. 2026 Aug 10;12(5):e211. doi: 10.1192/bjo.2026.12052 (PMC13458192; doi:10.1192/bjo.2026.12052)
Supplement: Supplementary file 1 [file S2056472426120523sup001.pdf]

Supplementary file 1: Steps describing the development of PEERS package

The following steps describe the process of development of the PEERS package in detail.

Step 1: Develop and translate the Peer Supervision Manual
Development of the PEERS app began with the drafting of the PEERS Supervision Manual. This manual was adapted from the supervision procedures outlined in the Counselling Relationship Manual (CRM) developed for the PREMIUM study (1,2). Key components included a clear definition of supervision, step-by-step guidance on delivering supervision and constructive feedback, and the creation of psychometrically sound rating scales to assess supervisor performance. The manual also addressed topics such as self-care and managing challenging interactions with participants. It underwent two rounds of review with input from expert collaborators.

Step 2: Develop and translate the PEERS App Content
The PEERS app was developed using CommCare, an open-source mobile platform created by Dimagi, a social impact tech company that specializes in building digital solutions for frontline health workers in low-resource settings . CommCare has been widely used in various interventions involving frontline workers in LMICs, for example, in an application for the management of childhood illnesses at primary health facilities in Burkina Faso (3); and in an m-health application used by community health workers for maternal, newborn and child health care services in rural Uttar Pradesh, India (4). In collaboration with Dimagi, the PEERS app was specifically designed to facilitate the delivery of the HAP and support NSPs' engagement in MBPS. The app development followed a structured, three-phase process (5). First, the team identified essential data points and workflows required to support HAP delivery and MBPS. Second, the team used an iterative approach to build wireframes, integrate workflow-based questionnaires, and incorporate local language content (Hindi) to ensure accessibility. Third, the team conducted internal testing of the app, during which technical issues were identified and resolved through a rigorous quality assurance process prior to broader user testing. The app's content and workflows were grounded in evidence-based protocols for HAP delivery, measurement-based care, and supervision. These components were co-developed by the study team and aligned with global standards for task-shared psychological interventions for depression. Following development, the app underwent beta testing, during which Dimagi's internal Quality Assurance team reviewed all workflows to ensure proper functionality and performance(5).

Step 3: Adapt the Peer Supervision Manual for PEERS App Training Materials
The content of the PEERS supervision manual was structured into a training course with five modules to support NSP training. This process involved developing the training guide, including the scripts for the videos, and translating into local languages when necessary. The scripts covered all the components of the PEERS supervision manual, such as understanding supervision, learning why supervision is important, and steps for engaging in MBPS using the PEERS app to register and schedule patients, conduct a treatment session, listen to session audio recordings, and submit ratings on the rating scales.

Step 4: Digitize the Peer Supervision Training Materials
This step involved creating digital training materials based on the course structure created in step 3 above, to support NSPs in learning the principles of MBPS and effective use of the PEERS app. The course was designed using slide-based presentations and instructional videos, which included filmed

demonstrations, voiceovers, and subtitles to enhance accessibility. This comprehensive training tool aims to strengthen NSPs' skills while ensuring consistent and accurate use of the digital platform. The training consists of five modules covering: the concept and types of supervision, core supervision skills, features of the PEERS app, and strategies for self-care. Each module includes multiple-choice quizzes to assess learner comprehension. Progress and quiz scores are tracked through the learning management system hosting the course, allowing for real-time monitoring of engagement and understanding.

Step 5: Pilot test and implement the PEERS app and supervision e-course

This step involved hands-on user testing with HAP-trained NSPs in Madhya Pradesh, India, including individuals with varying levels of smartphone experience. Between October 2021 and March 2022, NSPs participated in structured user-testing workshops that included app installation on different devices, functional testing through simulated real-world scenarios, and structured feedback collection via focus group discussions and individual interviews(5). The purpose of the user testing was to evaluate not only the app's technical performance but also its usability, clarity of workflows, and provider comfort with using smartphone-based tools. Feedback from this phase directly informed iterative improvements to the app's features and workflows. Each updated version incorporated input from both end users and expert collaborators. New releases were supported by training sessions that included a 'demo mode' with mock data, allowing providers to gain hands-on experience before using the PEERS app in real care and supervision contexts. This approach facilitated a smoother transition and encouraged confident adoption of each new version.

References

1. Chowdhary N, Anand A, Dimidjian S, Shinde S, Weobong B, Balaji M, et al. The healthy activity program lay counsellor delivered treatment for severe depression in India: Systematic development and randomised evaluation. British Journal of Psychiatry. 2016 Apr 1;208(4):381–8.
2. Patel V, Weobong B, Weiss HA, Anand A, Bhat B, Katti B, et al. The Healthy Activity Program (HAP), a lay counsellor-delivered brief psychological treatment for severe depression, in primary care in India: a randomised controlled trial. The Lancet. 2017 Jan 14;389(10065):176–85.
3. Blanchet K, Lewis JJ, Pozo-Martin F, Satouro A, Somda S, Ilboudo P, et al. A mixed methods protocol to evaluate the effect and cost-effectiveness of an Integrated electronic Diagnosis Approach (IeDA) for the management of childhood illnesses at primary health facilities in Burkina Faso. Implementation Science. 2016 Aug 4;11(1).
4. Prinja S, Nimesh R, Gupta A, Bahuguna P, Thakur JS, Gupta M, et al. Impact assessment and cost-effectiveness of m-health application used by community health workers for maternal, newborn and child health care services in rural Uttar Pradesh, India: A study protocol. Glob Health Action. 2016;9(1).
5. Singla DR, Fernandes L, Savel K, Shah A, Agrawal R, Bhan A, et al. Scaling up the task-sharing of psychological therapies: A formative study of the PEERS smartphone application for supervision and quality assurance in rural India. Global Mental Health. 2024 Feb 5;11.
