# Supplementary material for: Evaluating the costs of developing a digital supervision tool for task-shared depression care: findings from a multi-site study in India
Source: BJPsych Open. 2026 Aug 10;12(5):e211. doi: 10.1192/bjo.2026.12052 (PMC13458192; doi:10.1192/bjo.2026.12052)
Supplement: Supplementary file 2 [file S2056472426120523sup002.pdf]

Supplementary file 2
Template of survey tool for cost data collection

System Level Costs: Activity 1							
Time period: 12 months (MMYYYY-MMYYYY)							
The information will be collected monthly							
1. Human Resources							
1A. Human Resources for those directly involved in development (Salary and fringe benefits)							
**Involved Personnel**	**Role/Title**	**Responsibility**	**Time spent on this activity (months)**	**Proportion of FTE**	**Monthly Salary (incl. fringe benefits)**	**Travel allowance**	**Total costs**
1B. Human Resources for supervising the development (Salary and fringe benefits)							
**Name**	**Role/Title**	**Responsibility**	**Time spent on this activity (month)**	**Proportion of FTE**	**Monthly Salary (incl. fringe benefits)**	**Travel allowance, or reimbursement**	**Total costs**
2. Information technology							
2A. Using existing items							
**Items**	**Function**	**Quantity**	**Monthly rental price***	**Duration of the use (month)**	**% of time used for this activity**	**Total costs**	

2B. Buying new items								
**Items**	**Function**	**Quantity**	**Unit price**	**Life span (in years)**	**Yearly value**	**Duration of the use (months)**	**% of time used for this activity**	**Total costs**
2C. Software								
**Items**	**Function**	**Quantity**	**Price of subscription**	**Duration of subscription (in months)**	**Time used in this activity (%)**	**Total costs**		
3. Infrastructure support								
3A. Office space, utilities								
**Items**	**Monthly rental price or monthly bill**	**Total number of staff**	**# of staff for development**	**Duration of the use (month)**	**Total costs**			
Office space rental								
Internet connection								
Water								
Electricity								
3B. New office supply								

**Items**	**Quantity**	**Unit price**	**Life span (in years)**	**Yearly value**	**Duration of the use (month)**	**Deprecia tion rate**	**% of time used for this activity**	**Total costs**
3C. Existing office supply								
**Items**	**Quantity**	**Monthly price / rental cost**	**Duration of the use (month)**	**% of time used for this activity**	**Total costs**			
4. Community Support								
4A. Donated items								
**Items**	**Quantity**	**Monthly price / rental cost**	**Duration of the use (month)**	**% of time used for this activity**	**Total costs**			
4B. Interns								
**Person**	**Role/Title**	**Responsibility**	**Duration (months)**	**Proportion of FTE**	**Monthly Salary (incl. fringe benefits)***	**Total costs**		
Intern 1	Intern							
*: Cost of volunteer time: based on average salary if you need to hire someone to do the same job.								
4C. Community Volunteers								
**Person**	**Role/Title**	**Responsibility**	**Duration (days)**	**Stipend/Incentive Cost**				
Volunteer 1								
